# The Role of MSI Testing Methodology and Its Heterogeneity in Predicting Colorectal Cancer Immunotherapy Response

**DOI:** 10.3390/ijms26073420

**Published:** 2025-04-05

**Authors:** Alexandra Lebedeva, Anastasiia Taraskina, Tatiana Grigoreva, Ekaterina Belova, Olesya Kuznetsova, Daria Ivanilova, Anastasia Sergeeva, Alexandra Kavun, Egor Veselovsky, Vladislav Nikulin, Saida Aliyarova, Laima Belyaeva, Alexey Tryakin, Mikhail Fedyanin, Vladislav Mileyko, Maxim Ivanov

**Affiliations:** 1 OncoAtlas LLC, Leninskiy Prospekt, 4c1A, Office #1, Moscow 119049, Russia; lebedeva_a_a_1@staff.sechenov.ru (A.L.); taraskina@oncoatlas.ru (A.T.); grigoreva@oncoatlas.ru (T.G.); kuznetsova@oncoatlas.ru (O.K.); kavun@oncoatlas.ru (A.K.); egor.veselovsky@gmail.com (E.V.); aliyarova@oncoatlas.ru (S.A.); mileyko@oncoatlas.ru (V.M.); maxim.ivanov@oncoatlas.ru (M.I.); 2 Institute for Personalized Oncology, Sechenov First Moscow State Medical University, Trubetskaya Ulitsa, 8/2, Moscow 119048, Russia; laima@oncoatlas.ru; 3 Faculty of Physics, Lomonosov Moscow State University, Leninskiye Gory, 1, Moscow 119991, Russia; 4 Federal State Budgetary Institution N.N. Blokhin National Medical Research Center of Oncology, Kashirskoye Highway, 23, Moscow 115478, Russia; a.sergeeva1919@gmail.com (A.S.); nikulin@oncoatlas.ru (V.N.); atryakin@mail.ru (A.T.); fedianinmu@mail.ru (M.F.); 5 State Budgetary Institution of Health Care of the City of Moscow, Moscow Multidisciplinary Clinical Center “Kommunarka”, Department of Health of the City of Moscow, Ulitsa Sosenskiy Stan, 8c3, Moscow 142770, Russia; kravchuchek@gmail.com; 6 Department of Oncology, Pirogov Russian National Research Medical University, Ostrovityanova Ulitsa, 1, Moscow 117997, Russia

**Keywords:** colorectal cancer, liquid biopsy, next-generation sequencing, dMMR, MSI, immunotherapy

## Abstract

MSI is a crucial biomarker for selecting CRC patients for immunotherapy. Here, we analyze the first results from the observational prospective trial BLOOMSI (NCT06414304), which investigated the impact of MSI/dMMR testing methods and baseline tumor heterogeneity on treatment outcomes. Thirty MSI/dMMR+ CRC patients, who were candidates for immunotherapy, were enrolled. Depending on the local test used for MSI/dMMR, central PCR/IHC was performed. Baseline FFPE and liquid biopsy (LB) were analyzed with NGS. ORR (objective response rate) in the ITT population was 50% (95% CI, 31.3–68.7%). Concordance between local/central dMMR/MSI testing was 81%, and the concordance of IHC, PCR, NGS/FFPE, and NGS/LB was 68.4%. The ORR was similar for IHC+, PCR+, NGS/FFPE+, and NGS/LB+ patients (55.6%, 55.6%, 55%, and 57.9%, respectively). The ORR among patients with discordant IHC/PCR results was 0%, and the ORR among patients with NGS/LB-ORR was 25% (2/8 CR). Next, we performed quantitative MSI analysis, reflecting the clonality of MSI+ tumor cells. Multivariate analysis identified MSI clonality in FFPE (HR 0.63, 95% CI, 0.39–0.99, *p* = 0.0487) and LB (HR 3.05, 95% CI, 2.01–4.65, *p* < 0.00001) as independent predictors of progression. The ORR in patients with high clonality (≥7%, *n* = 4, NGS/LB) was 25%. We describe baseline methodological predictors of non-response to immunotherapy and propose a strategy for selecting potential non-responders. These findings warrant further investigation.

## 1. Introduction

Microsatellite instability (MSI) is the accumulation of mutations in short tandem repeats (STRs), or microsatellites, which consist of repeated 1–6 nucleotide sequences. Mutations in STRs typically result in changes in repeat length and reflect a deficiency in the mismatch repair (dMMR) system [1,2,3,4]. MSI is an important feature of colorectal cancer (CRC), presenting in approximately 15% of early-stage tumors and 4% of metastatic tumors [5,6], and plays an important role in the carcinogenesis of the CRC CMS1 subtype [7]. Driven by the emergence of immunotherapy in colorectal cancer treatment [8], MSI has gained a pivotal role in colorectal cancer treatment, leading to multiple approvals as a predictive biomarker [9].

The correct assessment of MSI status is critical for correct therapeutic decisions. The standard methods for assessing MSI and dMMR are polymerase chain reaction (PCR) and 4-antibody immunohistochemistry (IHC), respectively. These approaches are widely used in clinical practice and are recommended by professional consortia [6], notwithstanding the ongoing challenges [10]. Notably, MSI assessment by IHC has only indirect significance since the loss of function of the Lynch syndrome genes does not always lead to the development of MSI in the tumor, and there is also a possibility of false-positive IHC results, occurring in 5–11% of cases due to non-functional MMR proteins with a preserved antibody-binding capacity, the dislocation of functional MMR proteins in cells, focal losses, and other reasons [1,11]. Likewise, PCR-based MSI testing can also yield false-negative results, commonly due to low tumor cellularity or isolated MSH6 deficiency [12,13,14]. Next-generation sequencing (NGS) is a relatively new approach that allows for expanding the range of analyzed microsatellites to assess the MSI status and increase analytical sensitivity [6]. Due to the increasing demand for MSI tumor status testing, as well as the requirements for the truthfulness of results in all tumor types, new approaches to MSI testing have begun to be actively developed. The use of the NGS method allows for higher accuracy, the analysis of a wider range of analyzed microsatellites, as well as the simultaneous assessment of other clinically significant markers, such as high mutational load and alterations in target targets [6,15]. In addition, the use of NGS allows for the analysis of liquid biopsy, which is one of the solutions to the problem of the availability of tumor material, as well as monitoring the response to therapy [16,17].

In this paper, we present the first results of the BLOOMSI trial designed to observe the real-world practice of immunotherapy treatment of MSI/dMMR colorectal cancer with the aim of advancing the methodology of patient selection and therapy discontinuation. Here, we focus on the impact of the MSI assessment methodology prior to immunotherapy, how the MSI testing methodology affects treatment outcomes, and how to improve the methodology of patient selection for immunotherapy.

## 2. Results

### 2.1. Response to Treatment

**Figure 1:**
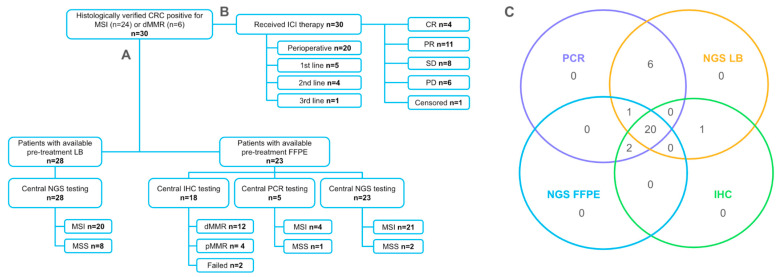
Study schema: (**A**) testing procedures; (**B**) treatment details; and (**C**) distribution of patients based on the different assays they received, showing how many patients were part of each assay group and how many received combinations of the assays. Patients with CRC were recruited based on the results of routine assessments of MSI/dMMR via gold-standard methods (IHC or PCR). Testing with an alternative gold-standard method (FFPE) and NGS (FFPE and/or LB) were performed centrally.

A total of 30 patients with CRC were included in this study. All patients had MSI/dMMR-positive tumors, as determined by a local routine assessment: PCR (*n* = 24) or IHC (*n* = 6). Most patients had right-sided tumors (56.7%) and non-metastatic disease (76.7%) (Table 1). All patients received immune checkpoint inhibitors preoperatively (*n* = 20, 66.7%) or as a systemic treatment for advanced disease (*n* = 10, 33.2%). Patients treated in the N.N.Blokhin Cancer Research Center (*n* = 20) received preoperative prolgolimab (1 mg/kg i.v. biweekly, up to 6 months) (*n* = 18, 90%) or 1st line treatment for advanced disease with either nivolumab monotherapy (*n* = 1, 5%) or combined with ipilimumab (*n* = 1, 5%). Patients treated in the Kommunarka Moscow Multidisciplinary Medical Center (*n* = 10) who were treated for advanced disease received nivolumab (*n* = 2, 20%), pembrolizumab (*n* = 5, 50%) or a combination of nivolumab and ipilumumab (*n* = 3, 30%); another two (20%) patients received preoperative nivolumab (*n* = 1, 10%) (Figure 1).

The ORR (objective response rate) in the ITT population was 50% (95% CI, 31.3–68.7%): 4 (13.3%) and 11 (36.7%) patients experienced complete (CR) and partial (PR) responses, respectively. Additionally, eight patients achieved stable disease (SD), resulting in a disease control rate (DCR) of 76.6% (95% CI, 57.8–90.1%). Patients receiving therapy preoperatively exhibited a radiological ORR of 60% (95% CI, 36–80.9%). Of those, patients who underwent surgical treatment (*n* = 16) demonstrated a pCR rate of 75% (95% CI, 47.6–92.7%). Among patients who received systemic therapy for advanced disease, the ORRs were 20% (95% CI, 5.1–71.6%) and 40% (95% CI, 5.3–85.3%) for the 1st and 2nd/3rd line therapy, respectively.

### 2.2. Agreement of Methods for MSI/dMMR Detection

**Figure 2:**
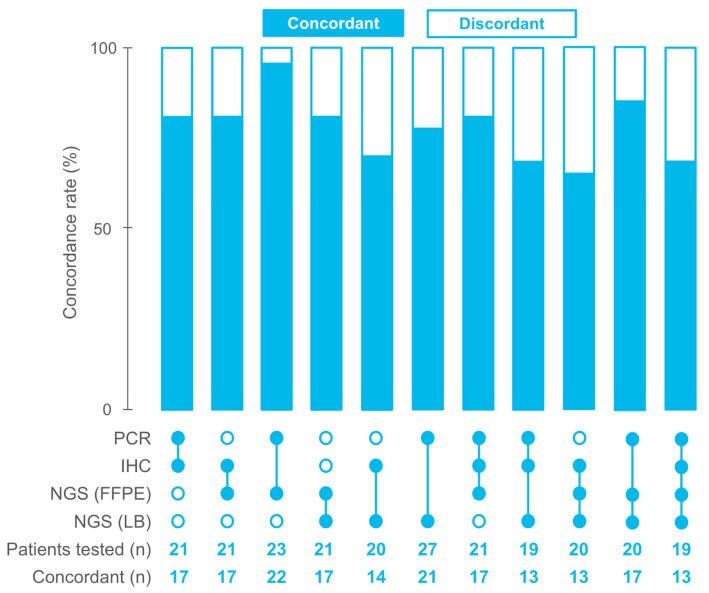
Concordance between the methods for MSI/dMMR detection used in this study.

After combining the local and central testing results, in total, 29 (96.7% of all patients) patients had PCR results, and 22 (73.3%) patients had IHC results. A total of 23 (76.7%) and 28 (93.3%) had results of NGS testing of FFPE and LB samples, respectively (Figure 1). Concordance between local and central dMMR/MSI testing (i.e., concordance between IHC and PCR) was at 81%. Although all patients included in this study had MSI-positive tumors, as determined locally by gold-standard methods (IHC or PCR), only 91.3% of available FFPE and 71.4% of LB samples were MSI-positive based on NGS. Among all the methods evaluated, the highest concordance was observed between PCR and NGS (FFPE), with a concordance rate of 95.6%. The concordance of IHC with NGS was low, with concordance rates of 81% and 70% for NGS (FFPE) and NGS (LB), respectively. NGS-based estimation of MSI in FFPE and LB samples was concordant in 80.1% of the cases. The agreement of all methods was observed for 17 FFPE samples and 13 FFPE + LB paired samples. The concordance rate of all four methods was 68.4% (Fleiss’s k = 0.2, indicating a slight agreement) (Figure 2).

### 2.3. Factors Influencing Treatment Outcomes

#### 2.3.1. Discordant MSI/dMMR Results

**Figure 3:**
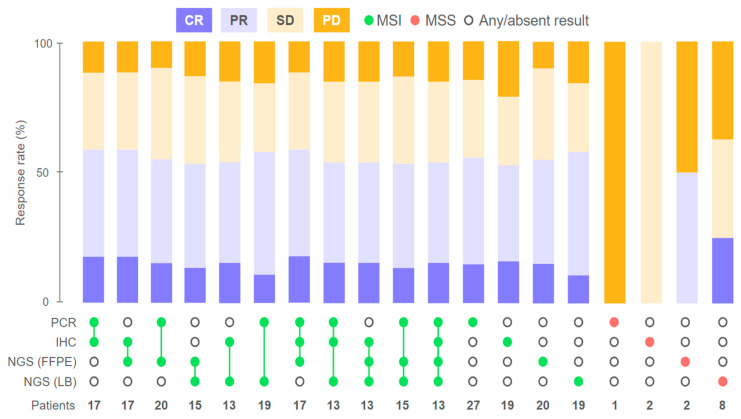
Response to treatment according to the MSI/dMMR testing result.

**Figure 4:**
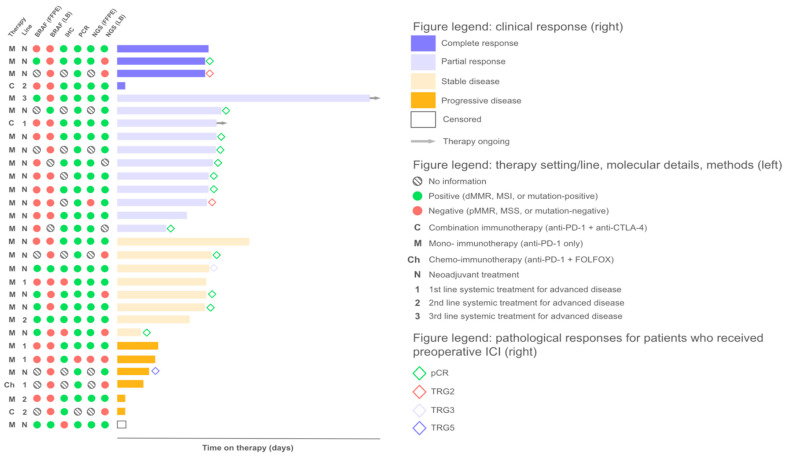
Treatment efficacy of ICI according to MSI/dMMR testing results and BRAF status. Swimmer plot of the duration of treatment (in days) for each patient (*n* = 30). Patients marked with arrows were still under treatment as of July 2024. On the left side of the figure, information on the therapy setting, BRAF mutational status, and MSI/dMMR testing results is given for each patient.

**Figure 5:**
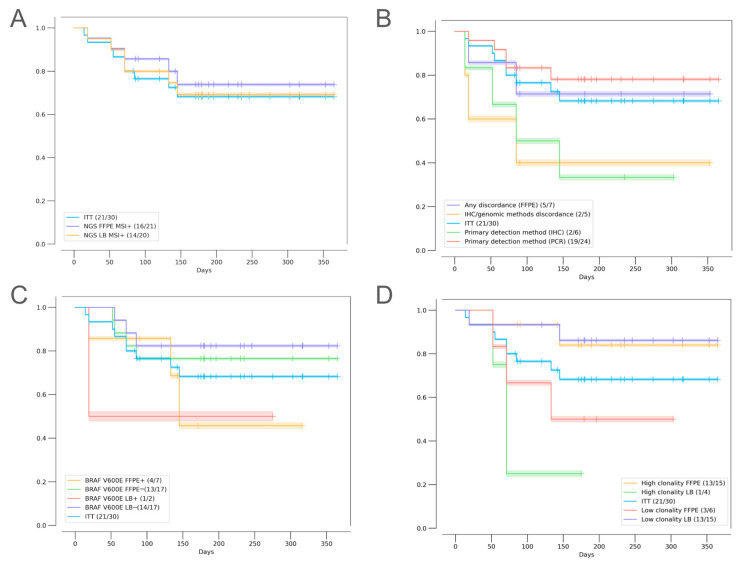
Kaplan–Meier estimates of progression-free survival in patients based on (**A**) MSI positivity in FFPE and LB (NGS); (**B**) discordant MSI/dMMR results and the primary method used for MSI/dMMR testing; (**C**) BRAF mutations found in FFPE and/or LB; and (**D**) MSI clonality values in FFPE and LB.

**Figure A1:**
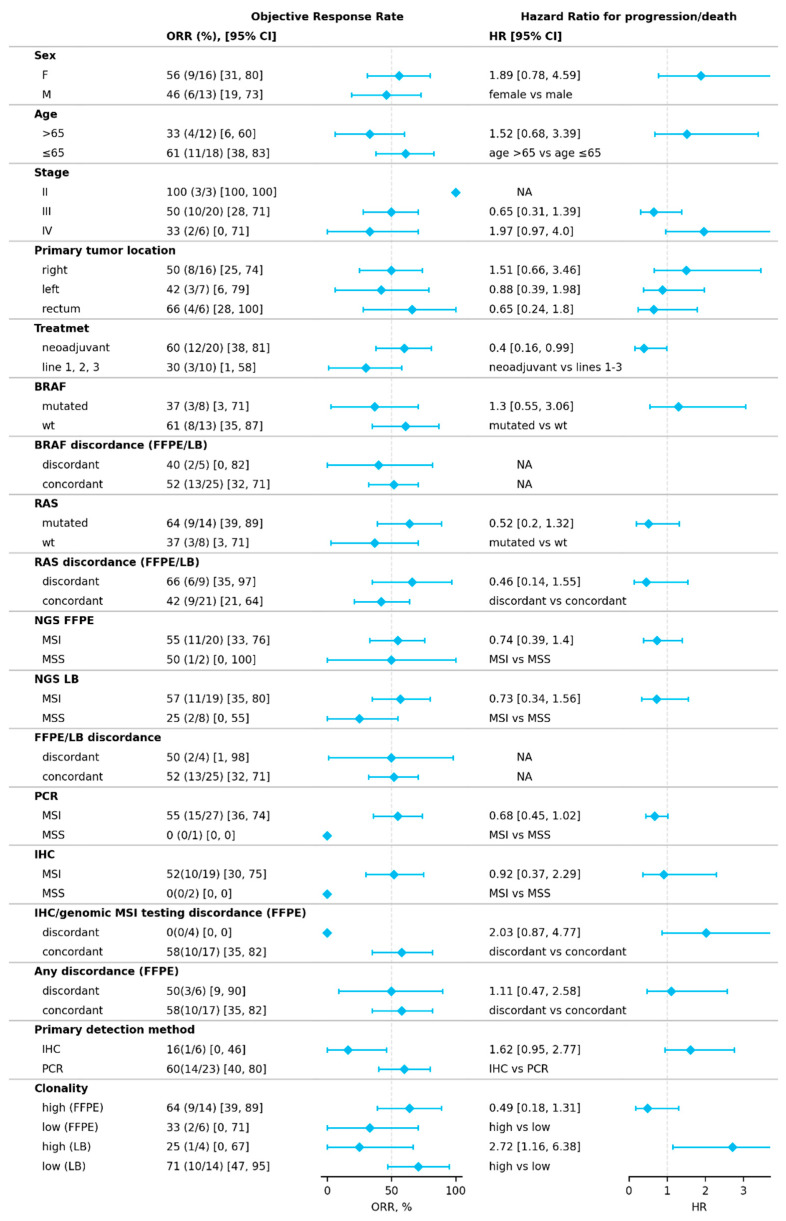
Subgroup analysis of objective response rate (**left**) and progression-free survival (**right**). The gray line in the left forest plot indicates the ORR in the intention-to-treat population.

First, we aimed to investigate whether any of the studied methods would be better at predicting treatment outcomes. We compared the IHC, PCR, and NGS results with the patients’ responses to therapy. The ORRs were 55.6% (14% CR, 40.6% PR), 55.6% (16.7% CR, 38.9% PR), 55% (15% CR, 40% PR), and 57.9% (10.5% CR, 47.4% PR) among patients with MSI/dMMR-positive status based on PCR, IHC, NGS (FFPE), and NGS (LB), respectively, and no statistically significant differences were observed between these four groups. The highest ORR of 62.5% was observed among patients whose tumor samples tested positive for MSI/dMMR with either both IHC and PCR or with IHC, PCR, and NGS (Figure 3, Figure 4, and Figure A1). The primary method used for local testing (PCR or IHC) did not play a significant role in the multivariate analysis, although patients initially tested with PCR demonstrated numerically longer progression-free survival (PFS) (Figure 5B and Table 2).

Intriguingly, objective responses were observed in 50% (1/2 PR) and 25% (2/8 CR) of patients with no MSI (i.e., microsatellite stability (MSS)) detected by NGS in FFPE and LB samples, respectively. Both patients with MSS LB who exhibited CR received preoperative ICI. One of these patients with dMMR/MSI based on IHC, PCR, and NGS (FFPE) had a BRAF p.V600E mutation identified in FFPE, but the latter was undetectable in LB, which could indicate a potentially low ctDNA fraction contributing to the MSS result. Another patient with MSS LB demonstrating a CR had a MSI-positive PCR result of local testing (central IHC and NGS (FFPE) were not performed due to the unavailability of FFPE). A patient who had a PR to preoperative ICI but had a MSS result following FFPE analysis based on NGS had low tumor purity, potentially causing a false-negative result since both PCR and LB analyses confirmed the MSI status (the patient’s FFPE sample also had an insufficient tumor cell content for IHC) (Figure 3 and Figure 4).

Noteworthy, in our patient cohort, a discrepancy in the MSI/dMMR status between IHC and genomic methods (PCR and NGS) had a negative influence on the ORR and PFS, as observed both in Kaplan–Meier plots and in the univariate analysis with no statistical significance (Figure 5B and Figure A1). Multivariate analysis demonstrated a statistically significant influence of this factor on patient outcomes (HR 2.16, 95% CI, 1.39–3.33, *p* = 0.0005) (Table 2). We identified five patients with discordant IHC and PCR/NGS results; of those, three patients had a pMMR status and two had dMMR as per IHC. The best response to ICI in this subgroup was SD (2/4); two patients had progressive disease (PD), and one patient was censored. These patients tended to be older (median age, 67 years vs. 54.5 years among patients who had concordant results of IHC and genomic methods), had stage III (66.7% vs. 63.5%) or IV (33.3% vs. 18.8%) CRC, and had tumors enriched for BRAF p.V600E mutations (60% vs. 35.7%). Additionally, these patients had a higher rate of LB MSS samples when compared to patients with consistent results of IHC and genomic methods (75% vs. 17.6%). Both patients with PD had MSS LB.

#### 2.3.2. MSI Clonality

To determine its role in mediating treatment benefits, we performed a quantitative analysis of MSI heterogeneity (MSI clonality) in samples with MSI-positive results based on NGS. When compared to patients with high MSI clonality in FFPE, patients with a quantitatively low level of MSI in FFPE tended to have a lower ORR and shorter PFS, while the opposite dynamics was observed for LB; however, statistical significance in univariate analysis was not reached (Figure A1 and Figure 5D). In patients with high clonal MSI, as detected in FFPE (clonality of 18% and higher), the ORR was 64% (9/14), compared to 33% (2/6) in patients with low clonal MSI (*p* > 0.05). In patients with high clonal MSI, as detected in LB (clonality of 7% and higher), the ORR was 25% (1/4), compared to 71% (10/14) in patients with low clonal MSI. Multivariate analysis confirmed that higher MSI clonality in FFPE was predictive of a lower risk of progression (HR 0.63, 95% CI, 0.39–0.99, *p* = 0.0487), while high MSI clonality in LB was associated with a higher risk of progression (HR 3.05, 95% CI, 2.01–4.65, *p* < 0.00001) (Table 2).

**Figure A2:**
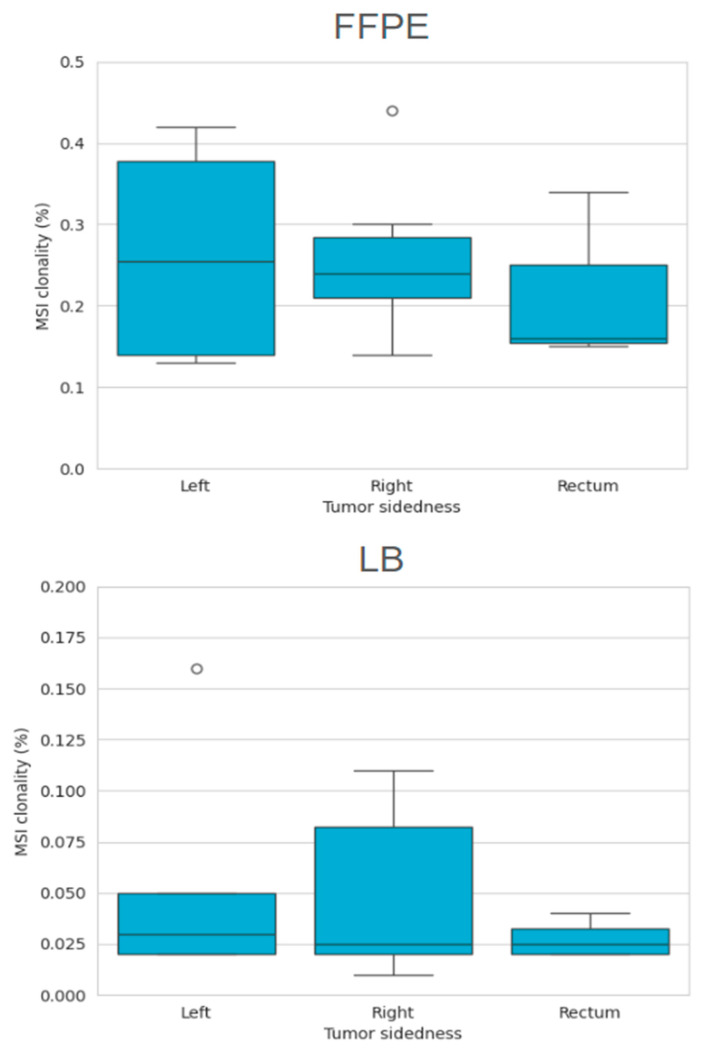
MSI clonality in FFPE and liquid biopsy (LB) samples based on tumor sidedness.

Interestingly, patients with right-sided tumors had a higher proportion of MSI with high clonality (*p* = 0.022) in the pre-treatment FFPE samples. No difference in MSI clonality distribution in LB MSI-positive samples was observed based on the primary tumor location (Figure A2). Although high MSI clonality was more frequent in right-sided tumors, no statistically significant differences in the patient outcomes depending on the primary tumor location were observed (Figure 5).

#### 2.3.3. Mutational Correlates

**Figure A3:**
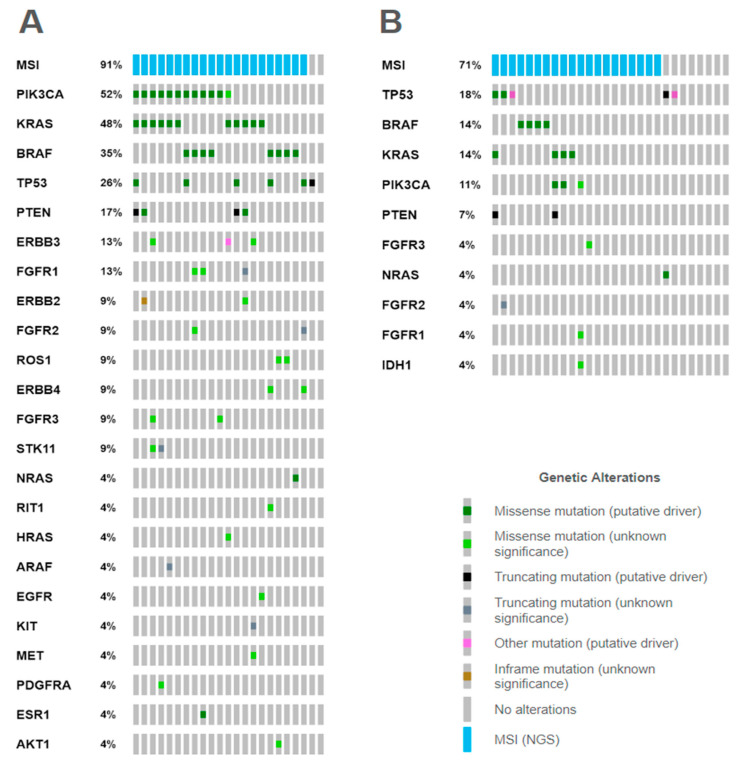
Oncoprints of somatic alterations identified following NGS analysis of the pre-treatment (**A**) FFPE and (**B**) liquid biopsy samples.

No statistically significant difference was observed between the frequencies of mutations in BRAF or TP53 genes in LB and FFPE. Meanwhile, KRAS and PIK3CA mutations were predominantly identified in FFPE samples (*p* = 0.029 and 0.021 for KRAS and PIK3CA, respectively). BRAF p.V600E mutations were observed exclusively in patients with confirmed MSI based on NGS in FFPE and/or LB (Figure A3). Patients with BRAF mutations exhibited a trend toward a lower ORR (37% vs. 61%) but not PFS, whereas patients with RAS mutations had a higher ORR and PFS. However, this did not reach statistical significance (Figure A1).

## 3. Discussion

In this paper, we describe the first results of the prospective observational BLOOMSI trial, focusing on the analysis of baseline FFPE and LB samples of MSI/dMMR-positive CRC patients. Specifically, we sought to determine whether discrepancies between different methods for MSI/dMMR detection, tumor heterogeneity related to quantitative and qualitative MSI assessment via NGS (MSI clonality), and the presence of mutations in key oncogenes in baseline samples could be used for selecting patients who are not likely to respond during immunotherapy treatment.

In this study, some discrepancies were observed between IHC, PCR, and NGS assessments of MSI/dMMR in FFPE samples. However, the ORR was not affected by the method when applied independently. The discordances in the results of FFPE testing, specifically with IHC, could be influenced by several factors [18]. For instance, as many as seven patients did not have available FFPE samples for central MSI/dMMR testing and two more patients had suboptimal sample quality for IHC. Additionally, as most patients in this study had received preoperative treatment, with only biopsy samples available for local and central MSI/dMMR testing, the limited tumor content may have rendered IHC results less informative [19,20]. In three cases, MSI was detected in FFPE but not in LB using NGS. All of these cases were early-stage CRC treated preoperatively. In these instances, BRAF mutations detected in FFPE were also absent in LB, suggesting a low circulating tumor DNA [21].

According to the literature, sources of discordant MSI/dMMR testing results might be related to interpretation errors or other biological factors. As previously mentioned, the pre-analytical phase is crucial for the correct diagnostic outcome of MSI/dMMR testing [18]. Furthermore, the misinterpretation of IHC results is reported as a major source of discordance, especially when not thoroughly reviewed [20]. The presence of somatic mutations in MMR genes has also been reported to influence the discordant MSI/dMMR testing result. Tumor heterogeneity, specifically the presence of subclonal MMR gene inactivation or heterogeneity in MLH1 promoter methylation, may also influence MSI/dMMR testing results. Finally, the presence of multiple primary tumors can also be a factor [22]. In our study, only one of the routine methods (IHC or PCR) was performed centrally, depending on the primary method used. Therefore, we cannot fully exclude the potential influence of pre-analytical factors or the incorrect interpretation of test results obtained locally. Furthermore, since the NGS analysis was prioritized over gold-standard testing when the FFPE samples were limited, the results of paired IHC/PCR were not available for all patients. Finally, neither MMR gene sequencing nor MLH1 gene methylation analysis was performed, limiting our ability to identify sources of discordance in our patient population.

It has been previously reported that the misdiagnosis of dMMR/MSI is a major contributor to the primary ICI resistance [23]. As per the study design, patients were included in this trial if they tested positive via IHC/PCR locally. We observed one patient with a potentially false-positive dMMR (IHC) result as both the PCR and NGS (FFPE and LB) methods found no evidence for MSI, exhibiting PD as the best response to ICI. Furthermore, in our study, we observed a notable lack of objective responses (with SD as the best response in this patient cohort) and shorter PFS among patients with discordant dMMR/MSI status as per IHC and the genomic methods (PCR and NGS). As previously mentioned, this study was not designed to allow a thorough analysis of sources of discordance, limiting our ability to identify the specific factors potentially associated with worse treatment outcomes. Multivariate analysis confirmed that the discrepancy between IHC and the genomic methods was an independent predictive variable for progression. Therefore, we argue that the use of both expression-based and genomic-based methods for dMMR/MSI detection may aid in selecting patients who are unlikely to respond to ICI.

In addition to the qualitative analysis of MSI, here, we raise the question of the importance of quantitative assessment. Intratumoral heterogeneity is a prominent contributor to therapeutic failure [24]. Previous studies have demonstrated that MSI exhibits temporal and spatial heterogeneity, which may decrease the sensitivity of MSI detection [25,26,27]. To estimate MSI heterogeneity, we calculated MSI clonality for every FFPE and LB sample identified as positive for MSI based on NGS. In the pre-treatment FFPE samples, higher MSI clonality was predictive of a lower risk of progression, while the opposite dynamics were observed for LB. Similar findings have been described for patients with EGFR-positive non-small cell lung cancer, where the clonal dominance of EGFR mutations was an independent factor of EGFR tyrosine kinase inhibitor benefit following an analysis of LB, and patients with clonal EGFR mutations exhibited longer PFS [28]. Therefore, for the first time, our findings suggest that the clonal architecture of MSI is a reliable predictor of treatment outcomes, both in FFPE and LB. However, previous studies have shown that single tissue samples can yield inconsistent results in the clonality analysis, justifying the use of paired LB-FFPE analysis [29]. This approach, similar to mutation-based assessments, can be used for patients with MSI-positive CRC to predict treatment outcomes.

The main limitation of our study is the small and heterogeneous patient cohort. Therefore, further validation of findings in randomized studies on larger patient cohorts prior to the incorporation of the results into clinical practice is warranted. Although all patients had MSI/dMMR-positive CRC, further prospective interventional studies with balanced clinical and morphological characteristics, as well as treatment settings, are needed to validate the findings. Another major limitation of our study is that the sequencing of MMR genes to determine which patients had Lynch syndrome was not performed as part of this study, which may have identified additional correlates with treatment outcomes and provided additional insights into the nature of MSI [30].

## 4. Materials and Methods

### 4.1. Study Design and Patients

Patients were recruited to the BLOOMSI trial, which evaluated the dynamics of MSI and genomic profiles in the course of immune checkpoint inhibitor treatment. BLOOMSI is a multicenter observational prospective trial (ClinicalTrials.gov number, NCT06414304). In this paper, we describe the analysis of the baseline samples (formalin-fixed paraffin-embedded (FFPE) and liquid biopsy (LB)) and analysis of the concordance of methods for MSI detection and their influence on therapy outcomes. The longitudinal analysis of serial LB samples will be described separately.

Patients with MSI/dMMR-positive CRC with measurable disease at any stage as per the Response Evaluation Criteria in Solid Tumor version 1.1 (RECIST v1.1) by local investigator/radiology assessment, Eastern Cooperative Oncology Group (ECOG) performance status of 0 or 1, and adequate organ function aged ≥18 years who were potential candidates for immune checkpoint inhibitor (ICI) treatment were eligible for this study. MSI/dMMR positivity had to be locally measured by a 4-antibody IHC assay or 5-loci PCR. After local MSI/dMMR testing, patients had to provide FFPE and baseline LB collected prior to the start of ICI for central assessment. Central testing had no impact on the decision to initiate and discontinue immunotherapy, and the results of central testing were shared with the treating physician and patient only after the completion of the immunotherapy treatment. Patients who had previously received ICI were not eligible for this study.

Patients were treated at the N.N.Blokhin Research Cancer Center (Moscow, Russia) or at the Moscow Multidisciplinary Clinical Center “Kommunarka” of the Department of Health of the City of Moscow (Moscow, Russia). Patients treated at the N.N.Blokhin Research Cancer Center for localized disease received prolgolimab, an anti-PD-1 monoclonal antibody [31], preoperatively as part of the clinical trial (ClinicalTrials.gov number, NCT06428487).

This study was approved by the ethical committee of the N.N.Blokhin Cancer Research Center. This study was conducted in accordance with the Declaration of Helsinki. All patients provided written informed consent prior to recruitment for this study.

### 4.2. Central MSI/dMMR Testing

Testing for dMMR/MSI using only one standard method was performed centrally, and the locally obtained results were used as is for further analysis. Patients whose tumors were MSI-positive based on PCR at inclusion underwent confirmatory IHC testing for dMMR with antibodies for MSH2 (G219-1129 and 271434-1B-3), PMS2 (A16-4 and 271434-1B-4), MLH1 (270868-1B-2), MSH6 (PU29 and 271434-1B-1), and positive/negative controls [32]. Patients whose tumors were dMMR-positive based on IHC at inclusion underwent confirmatory PCR testing for MSI (NR-21, BAT-26, BAT-25, NR-24, and NR-27). Central MSI/dMMR testing with PCR/IHC was performed on the pre-treatment FFPE samples. The results were interpreted in accordance with the European Society for Medical Oncology (ESMO) guidelines [6]. NGS-based MSI testing was performed on both FFPE and LB samples collected prior to treatment. Central testing for Lynch syndrome was not performed.

### 4.3. Next-Generation Sequencing

Central NGS was performed on the FFPE and LB samples collected prior to the start of ICI.

#### 4.3.1. DNA Extraction

Tumor DNA was isolated from archival FFPE blocks using the ExtractDNA FFPE kit (Evrogen JSC, Russia) according to the manufacturer’s protocol. Blood plasma samples were centrifuged for 15 min at 1900× *g* and 4 °C. The supernatants were collected and centrifuged for 10 min at 16,000× *g* and 4 °C and then stored at −80 °C. Circulating tumor DNA (ctDNA) from blood plasma was isolated using the QIAamp MinElute ccfDNA Midi Kit (Qiagen, Hilden, Germany).

#### 4.3.2. Library Preparation and Sequencing

Libraries were prepared using the Solo-test Driver panel (OncoAtlas, Moscow, Russia), covering 34 most commonly altered cancer-related genes and 39 STRs for MSI detection (53.8 Kb, 474 amplicons) using target enrichment technology. This panel is based on a multiplex PCR with two pools of primers. Amplicons were partially digested and ligated to universal adapters, purified on magnetic beads, and amplified with index primers. The index primers encode individual 8-base indices as well as an adaptor sequence, which can then bind to the flow cell (Illumina, San Diego, CA, USA or GeneMined, Changsha, China) through the adaptor sequence, and they can be identified through the sequencing of its unique index combination.

Quality control included the measurement of the concentration of the isolated DNA (≥0.5 ng/µL) and DNA libraries (≥0.6 ng/µL, corresponding to 4 nmol, with an average library length of 267 b.p.). Library concentrations were measured with a Fluo-200 fluorometer (AllSheng, Hangzhou, China) using a dsDNA HS Assay Kit (Thermo Fisher Scientific, Waltham, MA, USA). If the observed DNA concentration was low (0.5–1.5 ng/µL), the number of PCR cycles was increased to 21. If the library concentrations were low (0.3–0.6 ng/µL), the possibility of further sequencing was determined on a case-by-case basis with the consideration of the observed DNA concentration, as well as tumor purity and the proportion of necrosis determined by the central assessment of the initial sample by the pathologist. The target number of reads was 2.5 million, corresponding to an average coverage of 5000×. Libraries were sequenced on a Genolab M (GeneMind, Changsha, China) and FASTASeq300 (GeneMind, Changsha, China) at a read length of 2 × 150 bp.

#### 4.3.3. NGS Data Analysis

NGS data were processed using the “Solo AVES” web-based platform tailored for the analysis of sequencing data generated by the Solo-test Driver panel [33]. In brief, BWA [34] was used to align sequencing reads to the human genome GRCh37.p13 assembly. The SNV, MNV, and indel calling pipelines included (1) candidate gene variants calling with SiNVICT [35], STRELKA [36], and Mutect 2 [37]; (2) joining intersecting variations with in-house algorithms; (3) the collection of additional technical parameters of calling with Samtools and SGA [38]; (4) estimating variant calling probability versus a reference database of health tissue sequencing with an in-house algorithm modeling of site-specific noise level with poisson-beta distribution; and (5) final filtration of artifacts with ML-based modeling incorporating per-variant technical parameters collected on previous steps, along with prior probability of variant detection in specific tumor types. Copy number variation (CNV) calling was performed by employing multi-factor data normalization using a reference sequencing database of healthy tissue. Clinical interpretation of genomic variants was performed in accordance with community guidelines [39].

#### 4.3.4. MSI Analysis and Clonality Estimation

MSI analysis was performed using the estimation of the sum of k-mer prevalences corresponding to a shortened length by 2 b.p. and a longer length compared to reference STR sequences. MSI scores were calculated as the cumulative prevalence of k-mers corresponding to altered STRs across all STRs within a single sample [40]. Data analysis (including variant calling and MSI analysis) was conducted within the Solo AVES platform tailored to the Solo-test Driver panel and provided by the test kit’s supplier.

To estimate sample clonality, STR length distributions were compared to 400 reference (stable) samples. The linear transformation of the mean area above the reference distribution(s) was selected as the best approximation of clonality: 88.81 * s–11.42. For FFPE samples, clonality less than 18% was considered low, and for LB samples, values less than 7% were considered low.

### 4.4. Treatment Outcomes

Efficacy was assessed in the intention-to-treat (ITT) population, which consisted of all patients included in this study. Tumor response was assessed according to RECIST (version 1) by a treating physician. Disease progression was verified by imaging, performed locally at the treatment centers. The duration of treatment was calculated from the first to the last administration of treatment.

### 4.5. Statistical Analysis and Data Visualization

The objective response rate (ORR) to therapy between the groups was compared using Barnard’s test. The 95% confidence intervals (CIs) for the ORR were calculated using the Clopper–Pearson (exact) method. Other categorical values were tested using Fisher’s exact test and Barnard’s exact test. Concordance rates and Cohen’s and Fleiss’s kappas were used for the assessment of the concordance between methods for MSI/dMMR determination. Progression-free survival analysis was carried out using Weibull AFT regression, with the penalizer automatically selected based on the model’s AIC. The data were scaled beforehand. The analysis was carried out using Python v3.9.

## 5. Conclusions

We demonstrate here that the following methodological predictors may be associated with a lack of response to immunotherapy: (1) discordance between genomic and expression-based methods; (2) absence of MSI as determined by liquid biopsy; (3) low MSI clonality (15% and lower) in tumor tissue; and (4) high MSI clonality (7% and higher) in liquid biopsy. A composite score of two or more predictors, or extremely high MSI clonality in liquid biopsy alone (10% and higher), can identify a patient subgroup with an ORR of 0% (0/4, *p* = 0.06).

## Figures and Tables

**Table 1:** Clinical and demographic characteristics of the ITT population at baseline. * According to the results of central NGS testing.

Sex	Female	14 (46.7%)
	Male	16 (53.3%)
Median age (range), years	60.5 (28–85)	
Primary tumor location	Right	17 (56.7%)
	Left	7 (23.3%)
	Rectum	6 (20%)
Histology	Intestinal adenocarcinoma	27 (90%)
	Signet cell carcinoma	2 (6.7%)
	Mucinous adenocarcinoma	1 (3.3%)
Stage (AJCC)	II	3 (10%)
	III	21 (70%)
	IV	6 (20%)
T	T1	0 (0%)
	T2	0 (0%)
	T3	22 (73.3%)
	T4	8 (26.7%)
N	N0	6 (20%)
	N1	11 (36.7%)
	N2	13 (43.3%)
M	M0	23 (76.7%)
	M1	6 (20%)
	Mx	1 (3.3%)
Therapy	Prolgolimab	18 (60%)
	Pembrolizumab	5 (16.7%)
	Nivolumab	3 (10%)
	Nivolumab + Ipilimumab	3 (10%)
	Nivolumab + FOLFOX	1 (3.3%)
BRAF p.V600 (FFPE, NGS) *	Positive	8 (26.7%)
	Negative	15 (50%)
	Unknown	7 (23.3%)
RAS mutations (FFPE, NGS) *	Positive	14 (46.7%)
	Negative	9 (30%)
	Unknown	7 (23.3%)

**Table 2:** Multivariate analysis for progression-free survival. Abbreviations: CI—confidence interval, HR—hazard ratio, LB—liquid biopsy, and MSI—microsatellite instability. * Statistically significant interactions at *p* < 0.05.

Variable	HR (95% CI)	*p*-Value
MSI clonality (FFPE)	0.63 (0.39–0.99)	0.0487 *
MSI clonality (LB)	3.05 (2.01–4.65)	<0.00001 *
Discordance between IHC and genomic methods (present vs. absent)	2.16 (1.39–3.33)	0.0005 *
Age	3.69 (1.84–7.43)	0.0002 *
Sex (female vs. male)	1.12 (0.84–1.51)	0.4353
Metastatic disease (metastatic vs. localized)	0.76 (0.54–1.09)	0.1326
Primary method used for MSI detection (IHC vs. PCR)	1.08 (0.77–1.49)	0.666

## Data Availability

Data may be available upon reasonable request.

## Abbreviations

CI	confidence interval
CNV	copy number variation
CR	complete response
CRC	colorectal cancer
ctDNA	circulating tumor DNA
DCR	disease control rate
dMMR	mismatch repair deficiency
ECOG	Eastern Cooperative Oncology Group
ESMO	European Society for Medical Oncology
FFPE	formalin-fixed paraffin-embedded
FOLFOX	folinic acid (leucovorin, FOL), fluorouracil (5-FU, F), and oxaliplatin (Eloxatin, OX)
HR	hazard ratio
ICI	immune checkpoint inhibitor
IHC	immunohistochemistry
ITT	intention-to-treat (population)
LB	liquid biopsy
MSI	microsatellite instability
NGS	next-generation sequencing
ORR	objective response rate
PCR	polymerase chain reaction
PD	progressive disease
PFS	progression-free survival
PR	partial response
SD	stable disease
STR	short tandem repeats
